# Acute Foot Warming Enhances Plantar Tactile Sensitivity With Limited Postural Control Effects in Obese Older Adults

**DOI:** 10.1002/pri.70317

**Published:** 2026-08-17

**Authors:** Mathias Sosa Machado, Lissandra dos Santos Gonçalves, Amanda Carolina Paim Camponogara, Marieli Miranda Paz, Álvaro Sosa Machado, Felipe P. Carpes

**Affiliations:** ^1^ Applied Neuromechanics Research Group Federal University of Pampa Uruguaiana Rio Grande do Sul Brazil

**Keywords:** aging, foot, postural control, skin temperature

## Abstract

**Background and Purpose:**

Interventions to enhance tactile sensitivity, such as warming the feet, positively affect skin sensitivity with varying influence on postural control. Here, we hypothesize that foot warming may benefit tactile sensitivity and postural control in older adults, particularly those with obesity.

**Methods:**

Fifty participants divided into 16 middle‐aged adults (14 women, mean ± standard deviation: age 40.29 ± 3.73 years, body mass index, BMI, 24.51 ± 1.70 kg/m^2^), 17 non‐obese older adults (12 women, 72.71 ± 6.36 years, BMI 25.09 ± 2.84 kg/m^2^), and 17 obese older adults (13 women, 69.59 ± 7.19 years, BMI 32.65 ± 6.31 kg/m^2^) were evaluated for foot tactile sensitivity and center of pressure (CoP) displacement during natural and semi‐tandem stance before and after a warming protocol increasing foot skin temperature by approximately 6°C.

**Results:**

Acute foot warming consistently enhanced plantar tactile sensitivity across adulthood and aging, with the largest improvements observed in obese older adults. In contrast, effects on postural control were limited.

**Discussion:**

Consistent with previous studies, we found that foot warming acutely enhances plantar tactile sensitivity across adulthood and aging, with novelty and larger sensory benefits observed in obese older adults. However, improvements in postural control were not identified across different postural tasks. These findings identify foot warming as a simple strategy to acutely enhance plantar sensory function, particularly in older adults with obesity.

## 1 Introduction

Postural control deficits are a common concern in aging, partially explaining the higher risk of falls and loss of independence observed among older adults (Pitchai et al. 2019). Sensory information from peripheral mechanoreceptors, such as tactile sensitivity in the feet, and postural control are considered key components of balance in older adults (Bloem et al. 2000; Prieto et al. 1996). In this scenario, obese older adults are considered at higher risk of falls than matched‐age non‐obese individuals (Melzer and Oddsson 2016).

Comorbidities such as obesity are common among older adults and contribute to functional impairments, predominantly affecting the lower limbs (Vilaça et al. 2014), along with alterations in somatosensory functions (Andreato et al. 2020). It is well established that aging itself leads to reduced tactile function, and in this context, obesity acts as an aggravating factor, further diminishing plantar sensitivity. This double sensory impairment ultimately compromises postural stability. In situations with sensory deficits and a need for foot and ankle control, stimulating cutaneous feedback in the feet can enhance proprioception (Robb et al. 2025).

Sensory interventions have been proposed, especially considering the relevance of foot tactile sensitivity for movement control (Meyer et al. 2004; Nurse and Nigg 2001; Perry et al. 2000). Cooling or warming the foot sole by 5°C–6°C from baseline alters its mechanoreceptors' activity (Schlee et al. 2009). Warming of larger areas of the foot skin improves tactile sensitivity in young adults and non‐obese older adults, although the effects on postural control are variable (M. S. Machado et al. 2022, 2023). Considering that older adults tend to shift the CoP toward areas with higher sensitivity in the forefoot during standing (Á. S. Machado et al. 2016), interventions eliciting higher tactile sensitivity could emerge as promising for routine care in older populations, especially to deliver before physical training or rehabilitation sessions requiring postural control and confidence to perform the task.

However, it remains unclear whether an intervention that enhances tactile sensitivity might also alter postural control in individuals with conditions that affect multiple components of postural control, such as aging combined with obesity. To address this question, we investigated whether warming the feet enhances tactile sensitivity and improves postural control across different age groups, particularly in obese older adults. We hypothesize that warming the feet can enhance tactile sensitivity, and such an effect would positively impact postural control, particularly in obese older adults.

## 2 Methods

### 2.1 Study Design and Ethical Considerations

**FIGURE 1:**
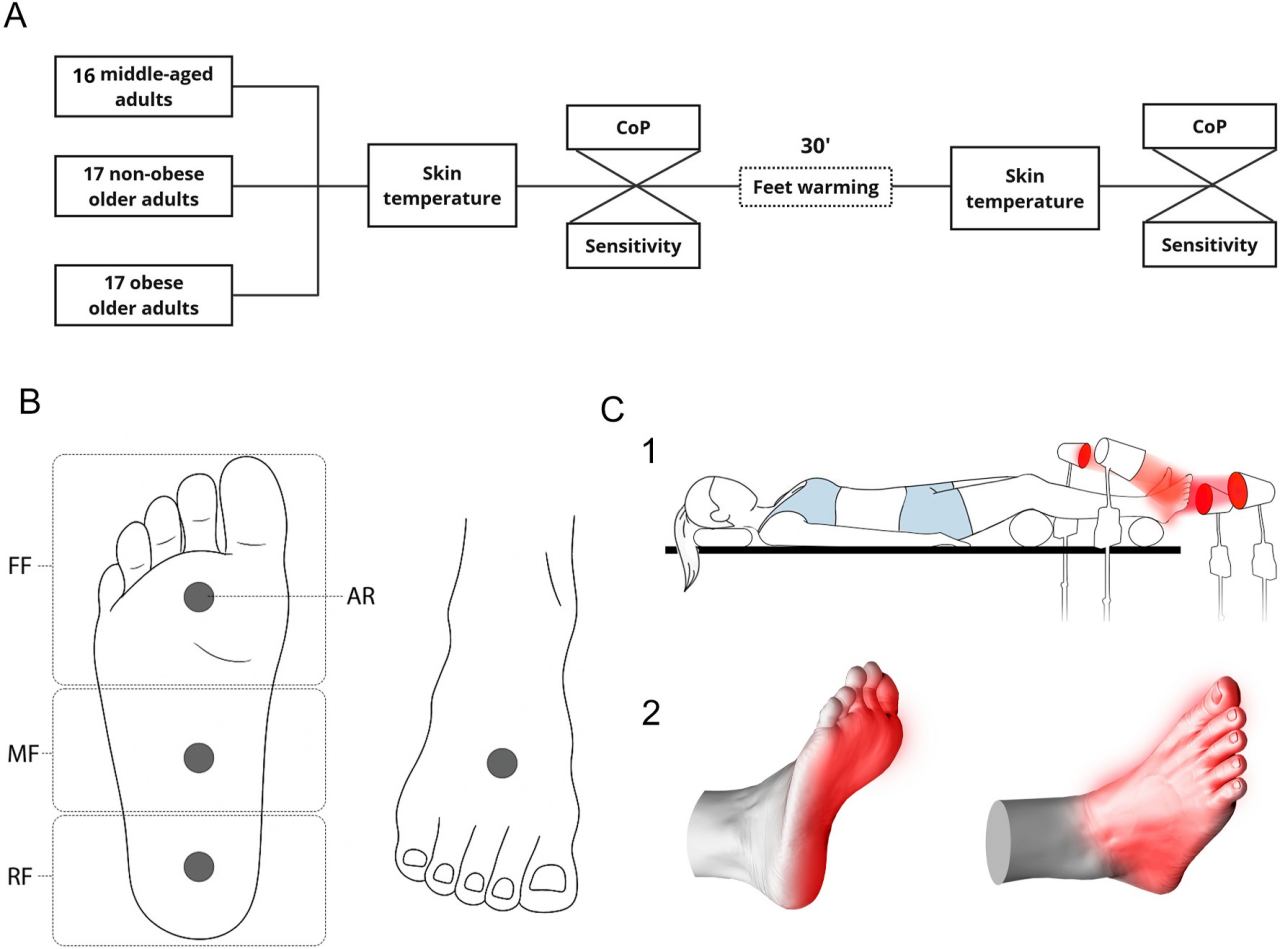
(A) Experimental design. (B) Gray circles indicate the regions where skin temperature was measured. FF, Forefoot; MF, Midfoot; RF, Rearfoot. (C) Foot warming protocol. (C1) Participant position with feet exposed to infrared radiation from four lamps. (C2) Illustration of the foot areas covered by the heating protocol.

Participants attended a single laboratory visit for assessment of foot cutaneous sensitivity and postural control before and after a foot warming protocol. Participants were instructed in advance to wear comfortable clothing that was neither tight around their legs nor rolled against the leg skin. They were instructed to avoid moderate or intense physical exercise, long walks, and new or unfamiliar footwear in the 24 h prior to testing, and to avoid very cold or hot baths and to refrain from applying creams, lotions, or any products to the skin of their feet. The evaluation room remained quiet, and the ambient temperature was standardized for all measurements (between 25°C and 26°C, 50%–60% air humidity). The season and time of day for assessments were consistent for all participants. Figure 1A illustrates the experimental design. All participants signed an informed consent form agreeing to participate in the study, which was approved by the local institutional review board for human research (IRB 73202423.8.0000.5323).

### 2.2 Participants

This study included 16 non‐obese middle‐aged adults (14 women, mean ± standard deviation: age 40.29 ± 3.63 years, body mass 65.1 ± 6.6 kg, height 1.63 ± 0.1 m, BMI 24.51 ± 1.70 kg/m^2^), 17 non‐obese older adults (12 women, 72.71 ± 6.36 years, body mass 62.0 ± 10.8 kg, height 1.57 ± 0.08 m, BMI 25.09 ± 2.84 kg/m^2^), and 17 obese older adults (13 women, 69.59 ± 7.19 years, body mass 82.1 ± 12.7 kg, height 1.59 ± 0.09 m, BMI 32.65 ± 6.31 kg/m^2^). To be eligible, volunteers should walk and stand independently, self‐report absence of visual or vestibular diseases, and report no lower limb injuries in the past 6 months. All participants were non‐fallers with no history of falls. Participants with a diagnosis of diabetes, Parkinson's, or Alzheimer's disease, as well as those engaged in regular physical training programs in the last 6 months or consuming controlled medications that might influence body balance, were not eligible. Furthermore, participants classified as overweight or with morbid obesity were not included. All participants were recruited from the local community through the distribution of flyers and digital cards. The sample size was estimated using GPower software (GPower 3.1.9.7, Franz Faul, University of Kiel, Germany) considering the MANOVA model with repeated measures between factors, with an effect size of 0.3, statistical power of 95% and alpha of 5%, suggesting the inclusion of 54 participants. To anticipate possible losses, 60 participants were recruited. Of these, 10 participants were excluded for having a body mass index classified as overweight in the non‐obese middle‐aged group (*n* = 1) or morbid obesity in the obese older adults (*n* = 6), not attending on the scheduled day and time (*n* = 2), and due to problems in signal processing after data collection (*n* = 1). Participants recruited were categorized into three groups based on age and body mass index (BMI, ratio body mass/height^2^). The middle‐aged adult group (*n* = 16, 14 women) included participants aged between 35 and 45 years with BMI between 18.5 and 24.9 kg/m^2^, corresponding to a normal BMI range and excluding obesity (WHO 2025). The non‐obese older adult group (*n* = 17, 12 women) included participants aged 65 years or older, with a BMI between 18.5 and 29.9 kg/m^2^, a range categorized below the threshold for obesity classification in older adults, and corresponding to the normal to overweight range excluding obesity (Kıskaç et al. 2022). The obese older adult group (*n* = 17, 13 women) included participants aged 65 or more with a BMI ranging from 30.0 to 39.9 kg/m^2^ and excluding morbid obesity, thereby establishing the obesity criteria for older adults (Kıskaç et al. 2022; Lipschitz 1994). Foot preference was determined by asking the preferred foot to kick a ball or start climbing stairs.

### 2.3 Foot Skin Temperature

After arriving at the laboratory, participants remained barefoot in a supine position on a stretcher for a 10‐min acclimatization period. Afterward, basal skin temperature was measured in different parts of the foot. Given that skin temperature is not expected to differ between feet (M. S. Machado et al. 2022), temperature was analyzed in four regions of the right foot (Figure 1B). Measures were conducted using a digital thermometer (TI‐550, Instrutherm) positioned approximately 1 m from the region of interest, with the lens aligned perpendicularly to the target surface (M. S. Machado et al. 2022).

### 2.4 Tactile Sensitivity

**FIGURE 2:**
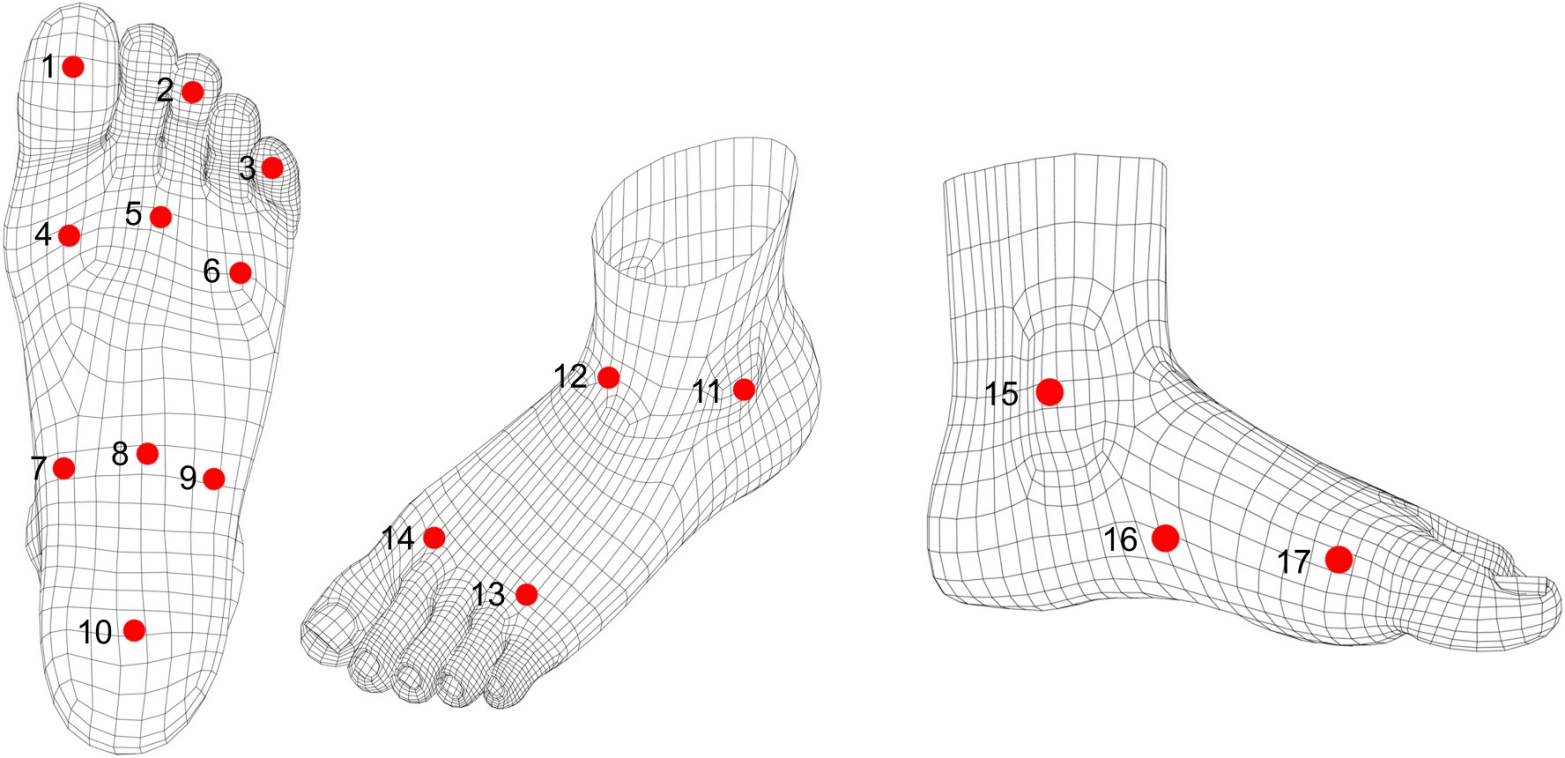
Anatomical sites where foot tactile sensitivity was assessed: 1, 2, 3, 4, 5, 6: forefoot; 7, 8, 9: midfoot; 10: rearfoot; 11: lateral ankle; 12: anterior ankle; 13, 14: dorsum; 15, 16, 17: medial aspect.

Considering that tactile sensitivity is not expected to differ between the right and left feet (Bueno et al. 2021), tactile skin sensitivity was measured only in the right foot using brand new Semmes‐Weinstein esthesiometers (Semmes‐Weinstein Monofilaments, San Jose, USA) (Bell‐Krotoski 1990). The esthesiometer consisted of six nylon filaments of equal length but varying diameters, producing a standardized pressure on the skin (Semmes‐Weinstein filaments ranging from 0.05 to 300 gf, later converted to log10 (Azevedo et al. 2020)), in accordance with the manufacturer’s calibration and usage recommendations. All participants were evaluated by the same examiner, who applied each monofilament perpendicularly to the foot skin with light and constant pressure until it began to bend. Seventeen sites were tested (see Figure 2 and Supporting Information S1), including the plantar surface, dorsum, lateral, and medial aspects of the foot (Hennig and Sterzing 2009). The stimuli were applied for approximately 1 second and repeated twice, ensuring that the filament buckled into a C‐shape. The participants were instructed to report where they perceived the tactile stimulus on their foot by pointing to the corresponding region in their hand. The tactile threshold was determined by applying progressively thicker filaments until the participant could detect and report the touch (Feng et al. 2009). The first monofilament detected by the participant was recorded as the tactile threshold. Any measurement involving filament slippage was disregarded and excluded from the analysis.

### 2.5 Postural Control

Postural control was assessed before and after the foot warming protocol. Participants performed quiet upright standing in (1) natural stance (feet parallel and spaced according to participant's preferences) and (2) semi‐tandem stance (non‐preferred foot placed slightly ahead of the contralateral foot). Participants were barefooted during all trials. In the natural stance, arms were relaxed beside the trunk; in the semi‐tandem stance, hands were placed on the hips. All tests were conducted in a quiet room, with adequate illumination and no visual disturbances. In both conditions, participants were instructed to fix their gaze on a visual target positioned at eye level, 3 m in front of them. Each trial lasted 30 seconds, followed by a 30‐s rest period. The order of stance conditions was randomized across participants.

CoP data were recorded at 100 Hz using a three‐dimensional force plate embedded at the ground level (OR6 2000, Advanced Mechanical Technology Inc., Watertown, MA, USA) and low‐pass filtered at 10 Hz with a 4th order Butterworth filter. A layer of ethylene vinyl acetate (2.0 mm thick) was placed over the force plate surface to prevent heat exchange between the foot and the force platform surface. The mean of three trials for each stance condition was used for further analysis of the following CoP outcomes: (a) 95% confidence ellipse area of the CoP (CoP area, in cm^2^), (b) mean resultant CoP velocity (CoP velocity, in cm/s), and (c) CoP amplitude in the anteroposterior and mediolateral directions (AmpAP and AmpML, respectively, in cm) (Prieto et al. 1996).

### 2.6 Warming Protocol

The warming protocol used infrared light to increase foot skin temperature by approximately 6°C (Schlee et al. 2009). Participants lay supine on a stretcher and were instructed to relax for 10‐min acclimation period. The foot and ankle were accessible, avoiding contact with the stretcher or clothes. Four infrared lamps (Philips PAR38, 150 W–230 V) were positioned 35 cm from the skin surface targeting the plantar surface of the foot and the entire dorsal foot and ankle region. The foot warming protocol lasted 30 minutes, with no intervals or reheating (M. S. Machado et al. 2023). Participants were randomly assigned to the order of the postural control and tactile sensitivity assessments, both conducted before and after the foot‐warming protocol. Once the temperature increase was achieved (in about 30 min, as reported in previous studies (M. S. Machado et al. 2023; Schmidt et al. 2016)), postural control and tactile sensitivity assessments were conducted immediately. The fluence delivered to the participants’ feet was estimated at 270 J/cm^2^, following dose‐response principles, calculated using the formula $\text{Dose}\;\left(J/{\text{cm}}^{2}\right)=\text{Irradiance}\;\left(\text{mW}/cm^{2}\right)\times \text{time}\;\left(s\right)\times 0.001$ (Hamblin 2017; Huang et al. 2009). To prevent thermal discomfort, the warming protocol adhered to safety guidelines on thermal comfort in footwear environments (ISO 2011). The protocol aligns with protocols used to evaluate thermal stimulation of the foot (Schlee et al. 2009; Schmidt et al. 2016). Figure 1C illustrates the foot warming protocol.

### 2.7 Statistical Analysis

Data normality was assessed using Shapiro–Wilk test. For non‐parametric demographic variables, Kruskal–Wallis test was used to compare groups. For demographic variables that followed a normal distribution, one‐way Analysis of Variance (ANOVA) was used to compare the groups. Tactile sensitivity changes were analyzed through a mixed‐model ANOVA, with region (17 sites) and time (2 points) as within‐ and‐subjects factors, and group as a between‐subjects factor. Repeated‐measures ANOVA was used to compare skin temperature between groups (middle‐aged adults, non‐obese older adults, and obese older adults) across time (pre‐ and post‐intervention) and foot regions (forefoot, midfoot, rearfoot, and dorsum). A mixed‐design MANOVA was applied to analyze CoP outcomes, entering Group as a between‐subject factor and time as a within‐subject factor. Univariate follow‐ups extracted pooled residual error terms from the repeated‐measures structure. This analysis followed pairwise comparisons adjusted using the Bonferroni method. Effect sizes were reported as partial eta squared (*η* ^2^ *p*). All statistical analyses were performed using the Statistical Package for the Social Sciences (SPSS) Version 27.0.1 (IBM Corp., Armonk, NY, USA), with the significance level set at 0.05.

## 3 Results

Baseline foot temperature (see Supporting Informations S2 and S3 for detailed results) did not differ between groups [Wilks' lambda = 0.891; *F*_(2, 15)_ = 0.918; *p* > 0.05]. The foot warming protocol increased temperature across all foot regions and in all groups [Wilks' lambda = 0.012; *F*_(1, 16)_ = 1375.159; *p* < 0.001]. The mean temperature increase after the warming protocol was 7.0 ± 0.5°C for middle‐aged adults, 6.9 ± 0.4°C for non‐obese older adults, and 7.0 ± 0.8°C for obese older adults.

**FIGURE 3:**
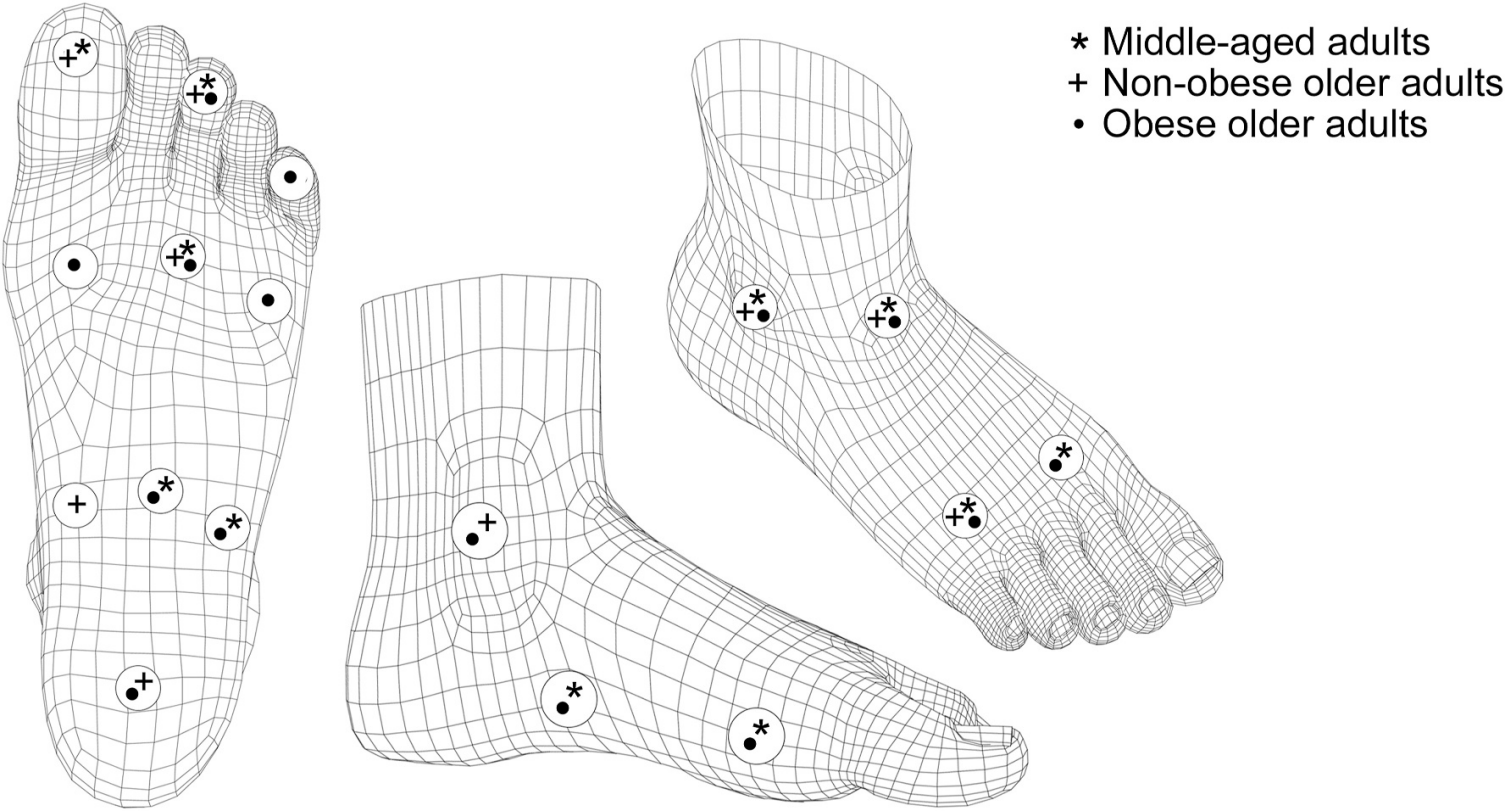
Identification of the sites in which tactile sensitivity increased after the heating protocol across the different groups. Complete sensitivity data are available in the Supporting Information S1.

Foot warming increased foot tactile sensitivity in all groups (Figure 3). Tactile sensitivity differed between groups at both pre‐ and post‐intervention times [F_(2,47)_ = 88.493, *p* < 0.001], and between pre‐ and post‐intervention assessments within each group [F_(1, 47)_ = 32.875, *p* < 0.001].

**FIGURE 4:**
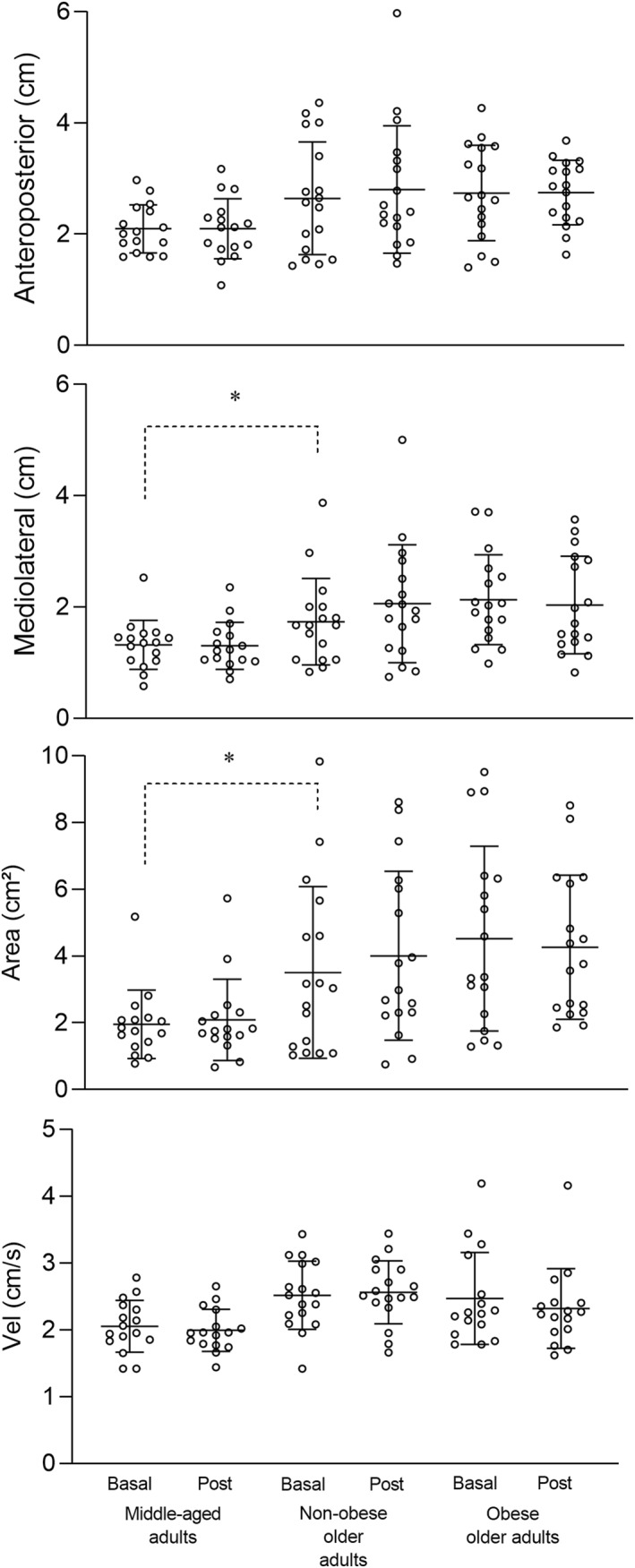
Mean, standard deviation, and individual responses of CoP to the heating protocol in the natural stance. Anteroposterior: amplitude of center of pressure displacement in the anteroposterior direction; Mediolateral: amplitude of center of pressure displacement in the mediolateral direction; Area: 95% prediction of the ellipse area;Vel: mean displacement velocity of the center of pressure.

CoP outcomes during natural stance (Figure 4) at baseline differed between groups (Pillai’s Trace = 0.316, *F*_(8, 90)_ = 2.110, *p* = 0.043, *η* ^2^ *p* = 0.158). Univariate follow‐up analyses revealed that obese older adults presented greater mediolateral amplitude [*F*_(2, 47)_ = 5.575, *p* = 0.007, *η* ^2^ *p* = 0.192] and CoP area [*F*_(2, 47)_ = 5.293, *p* = 0.008, *η* ^2^ *p* = 0.184] than middle‐aged adults. For the semi‐tandem stance at baseline, there was a significant multivariate difference between groups (Pillai’s Trace = 0.380, *F*_(8, 90)_ = 2.638, *p* = 0.012, *η* ^2^ *p* = 0.190). Univariate analyses showed that obese older adults presented greater in mediolateral amplitude compared with middle‐aged adults [*F*_(2, 47)_ = 3.335, *p* = 0.044, *η* ^2^ *p* = 0.124] and non‐obese older adults exhibited greater CoP velocity compared with middle‐aged adults [*F*_(2, 47)_ = 5.138, *p* = 0.010, *η* ^2^ *p* = 0.179]. No significant differences were observed for anteroposterior amplitude (*p* = 0.353) or CoP area (*p* = 0.193).

Considering both group and time effects on CoP outcomes during natural stance, the multivariate analysis did not reveal a significant main effect of group (Pillai’s Trace = 0.269, *F*_(8, 90)_ = 1.750, *p* = 0.098, *η* ^2^ *p* = 0.135) or time (Pillai’s Trace = 0.026, *F*_(8, 90)_ = 0.288, *p* = 0.884, *η* ^2^ *p* = 0.026). However, exploratory univariate follow‐up analyses suggested that obese older adults had greater anteroposterior amplitude [*F*_(2, 47)_ = 4.067, *p* = 0.023, *η* ^2^ *p* = 0.148], mediolateral amplitude [*F*_(2, 47)_ = 5.453, *p* = 0.007, *η* ^2^ *p* = 0.188] and CoP area [*F*_(2, 47)_ = 5.711, *p* = 0.006, *η* ^2^ *p* = 0.196] than middle‐aged adults. No significant between‐group differences were observed for CoP velocity.

**FIGURE 5:**
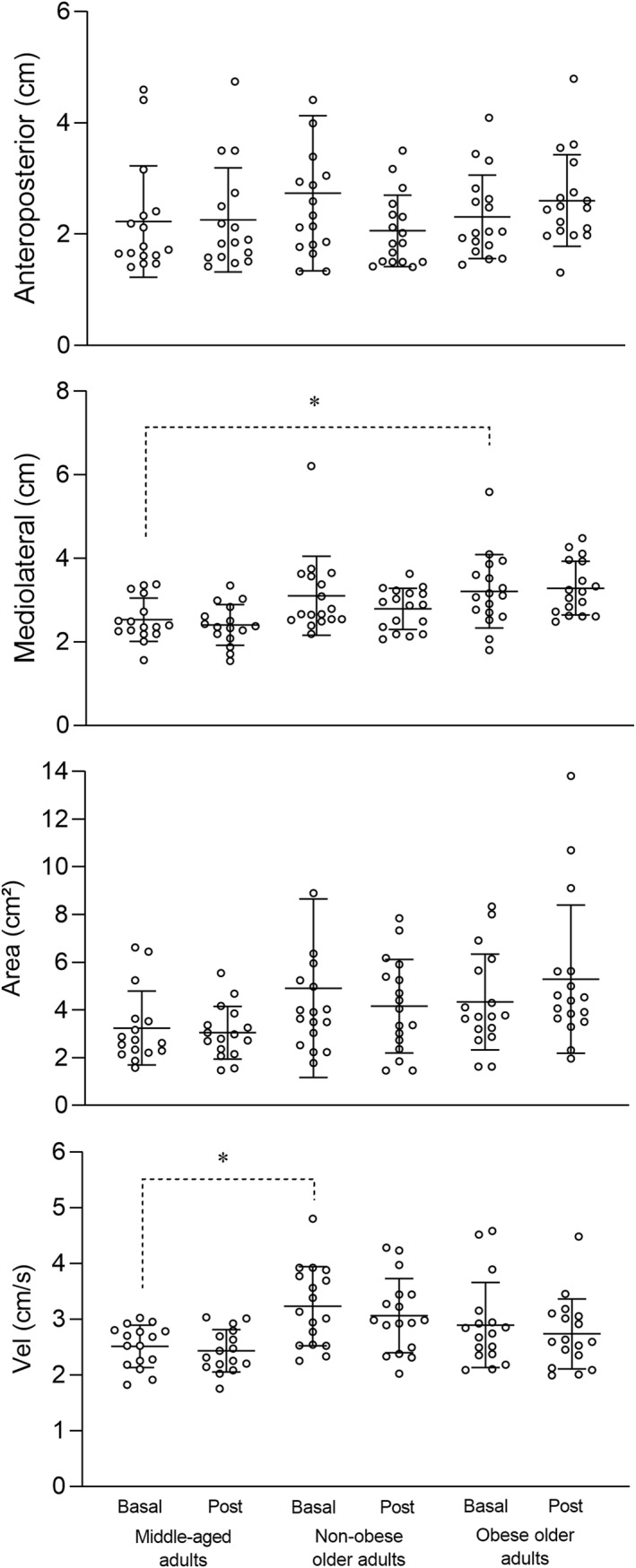
Mean, standard deviation and individual responses of CoP to the heating protocol in the semi‐tandem stance. Anteroposterior: amplitude of center of pressure displacement in the anteroposterior direction; Mediolateral: amplitude of center of pressure displacement in the mediolateral direction; Area: 95% prediction of the ellipse area;Vel: mean displacement velocity of the center of pressure.

Considering both group and time effects on CoP outcomes during semi‐tandem stance (Figure 5), despite no time effect (Pillai’s Trace = 0.162, *F*_(8, 90)_ = 2.132, *p* = 0.093, *η* ^2^ *p* = 0.162), a main effect of group was observed (Pillai’s Trace = 0.493, *F*_(8, 90)_ = 3.683, *p* = 0.001, *η* ^2^ *p* = 0.247). Univariate follow‐up analyses revealed that obese older adults exhibited greater mediolateral amplitude compared with middle‐aged adults [*F*_(2, 47)_ = 6.743, *p* = 0.003, *η* ^2^ *p* = 0.223], whereas non‐obese adults showed greater CoP velocity compared with middle‐aged adults [*F*_(2, 47)_ = 5.493, *p* = 0.007, *η* ^2^ *p* = 0.189]. No between‐group differences were observed for anteroposterior amplitude [*F*_(2, 47)_ = 0.369, *p* = 0.694] or CoP area [*F*_(2, 47)_ = 2.885, *p* = 0.066]. Although no significant multivariate main effect of time was observed for the semi‐tandem, exploratory univariate analyses indicated a significant reduction in CoP velocity from baseline to follow‐up [*F*_(1, 47)_ = 7.446, *p* = 0.009, *η* ^2^ *p* = 0.137].

## 4 Discussion

In this study, we investigated whether foot warming improves tactile sensitivity and postural control in middle‐aged adults, non‐obese older adults, and obese older adults. We hypothesized that warming the feet could enhance tactile sensitivity, and such an effect would positively impact postural control, particularly in obese older adults. In line with our initial hypothesis, important effects of the intervention on tactile sensitivity were identified. The warming protocol led to improvements in tactile sensitivity across all groups, particularly among obese older adults. In contrast, effects on postural control were mostly non‐significant.

Foot warming enhanced tactile sensitivity in all three groups. In a previous study, warming foot skin temperature by about 6°C also enhanced the tactile thresholds in the foot (Schlee et al. 2009). Our results support the reproducibility and consistency of this warming protocol to enhance tactile sensitivity, supporting the relationship between skin temperature and sensorial responses. Mechanical stimuli are primarily detected by mechanoreceptors such as Vater‐Pacini corpuscles, located in the deeper skin layers, and Merkel discs, found in the more superficial layers (R. S. Johansson and Vallbo 1983). The function of Vater‐Pacini corpuscles may exhibit reduced refractory period duration during action potential generation due to warming (Ishiko and Loewenstein 1961). This may explain the increased tactile sensitivity observed. In addition, obese older adults may experience reduced blood flow and low peripheral temperature in extremities due to vascular impairments (Limberg et al. 2016), which negatively affect mechanoreceptor function (Grosskreutz et al. 1999, 2000). Considering that infrared radiation has a vasodilative effect and tends to increase peripheral blood flow (Ise et al. 1987), a potential pathway for tactile enhancement may involve increased perfusion of free nerve endings (Ishiguro et al. 2010; Tsai and Hamblin 2017). This rationale could explain the broader increase observed among obese older adults, but further research is needed. Linked to this, surrounding temperature elevation via increased blood flow may also exert thermal effects on mechanoreceptors containing temperature‐dependent enzymes in their inner core, thereby improving the efficiency of tactile stimulus transduction (Watanabe and Ide 1986). The hypothesis that changes in blood flow can also contribute to improvements in sensitivity requires further experimental investigation.

Non‐obese and obese older adults exhibited improvements in tactile sensitivity across more sites (i.e., larger areas) than middle‐aged adults. Interestingly, improvements in sensitivity were observed in the heel, toes and fifth metatarsal regions following warming, which is an important finding for individuals experiencing sensory deficits in the rearfoot compared to the forefoot (Á. S. Machado et al. 2016). Increasing the availability of sensory information in younger adults may be difficult, given that their sensory systems are likely to be largely intact. This may explain why fewer regions showed improvements in tactile sensitivity following the warming protocol in the middle‐aged adults, and why the impact of warming on postural control was not substantial. In our study, this group was between 35 and 45 years old, an age span known to involve the onset of natural dysfunction in cutaneous receptors (García‐Piqueras et al. 2019), which may explain the slight improvements observed.

The consistency of the warming effects observed for tactile sensitivity was not reflected in the quiet upright standing postural control tasks. Postural control during standing results from a combination of sensorimotor information of both internal and external origin (Bouisset and Zattara 1990) and involves redundancies that enable improved control of body sway (Santos et al. 2010). In our study, we chose not to manipulate vision, which is considered to be the most critical source of sensory information (Giagazoglou et al. 2009). This methodological decision was based on the fact that removing visual input could present an additional challenge, making it difficult for individuals to complete the postural task (Poulain and Giraudet 2008). Given these choices, it is important to note that in the adult and older adult groups, CoP results did not differ before and after the intervention. Previous studies with young adults (M. S. Machado et al. 2022) and older adults (M. S. Machado et al. 2023) also demonstrated that after this warming protocol, the CoP responses are highly variable between individuals. Therefore, in this study, we included an additional postural task that, by reducing the base of support, could impose more difficulty on the individuals, especially the older ones.

Challenging postures seem to increase the demand for afferent information. Considering that the cutaneous sensitivity and proprioception around the ankle joint play a key role in balance (Lopes et al. 2014) and position sense (H. Johansson 1991; Wu et al. 2015), it is plausible that during the semi‐tandem task, the foot dorsiflexion and plantarflexion postures induce skin stretching and wrinkling. These skin deformations are relevant to the activation of Merkel and Ruffini receptors. Heat exposure in the ankle region may have potentiated these receptors’ response to such deformations, and this enhancement in sensory input may help explain the subtle improvements during the semi‐tandem stance. Since the multivariate test for the effect of time did not reach significance and the *p*‐value for the interaction in velocity was 0.009 with a small effect size, the observed reduction in CoP velocity should be described as an exploratory finding interpreted as hypothesis‐generating rather than confirmatory. In any case, a small signal of effect may be particularly relevant for older adults, as CoP velocity reflects postural control strategies adopted to prevent falls (Prieto et al. 1996). However, these findings require further investigation, particularly through experimental designs that incorporate multiple metrics to assess postural control or manipulations of the postural task, perhaps to explore changes in reactive responses following increased plantar tactile sensitivity.

Our study has inherent limitations. The semi‐tandem stance was considered a challenging condition for evaluating postural control in older adults. However, it remains to be investigated whether the contributions of foot warming to postural control increase with the complexity of the postural task. The absence of a control group is another limitation. While establishing a constant foot temperature without sensory stimulation is technically challenging, the lack of a control condition limits our ability to isolate the specific effects of infrared radiation from those of prolonged rest. Future studies should incorporate a sham intervention (e.g., using a non‐heating lamp) to further clarify the causal mechanisms behind the observed results. Participants remained supine for approximately 40 min to complete the acclimatization period and warming protocol, which could influence arousal levels or muscle tone independently of the thermal stimulus. A stabilization period was provided to minimize acute instability or blood pooling. While our observation of the participants' behavior during the test does not indicate such an effect, future studies could incorporate a longer stabilization phase or monitor blood pressure to ensure that orthostatic adjustments do not confound the sensorimotor responses. Finally, the role of body mass as a mechanical confounder must be considered. Heavier individuals naturally experience gravitational torques during standing, which can lead to larger CoP excursions (Chow et al. 2006). In our study, obese older adults had significantly higher body mass than non‐obese older adults, which likely contributed to the baseline differences. While the weight difference between our middle‐aged and non‐obese older groups was negligible (∼3 kg), the mechanical load in the obese group represents an inherent factor that is methodologically difficult to isolate from the biological effects of obesity on postural control and must be considered.

## 5 Implications for Physiotherapy Practice

The findings suggest that warming the feet is a strategy to acutely enhance tactile sensitivity, which is often reduced with age. Middle‐aged adults experience a transient decline in tactile perceptual function (García‐Piqueras et al. 2019). In our study, we expected non‐older adults to show little improvement in sensory function and postural control, but this was not observed in tactile function. This finding opens possibilities for treating individuals who are not older adults but still experience peripheral sensory deficits. Regarding postural dynamics, the effects of foot warming were not clearly identified, and the implications of the subtle reduction in CoP velocity during the challenging semi‐tandem stance in obese older adults should be interpreted with caution. This particular variation emerged as a strictly exploratory, hypothesis‐generating trend, a secondary finding within a globally non‐significant multivariate model. Therefore, this slight modulation suggests a potential physiological trend that warrants further investigation.

Physiotherapists should take into account that peripheral temperature plays an important role in patient assessment and treatment. Improvements in tactile cutaneous perception through warming could optimize sensorimotor integration, thereby facilitating lower‐limb reflex pathways (Howe et al. 2023). Although our findings involve independent community‐dwelling adults, future research could investigate whether enhancing sensory feedback through the somatosensory system helps restore functional capacity in patients during early‐stage rehabilitation. Future research should explore the mechanisms involved in reducing the perceptual threshold in the feet and whether different stability assessment methods may show more precise changes.

## Funding

This work was supported by funding from the Brazilian National Council for Scientific and Technological Development (CNPq), granted to M.S.M., A.S.M., A.C.P.C., and F.P.C. (Grant 306401/2022‐3). M.M.P. received support from the Coordination for the Improvement of Higher Education Personnel (CAPES), Finance Code 001. L.S.G. was supported by PDA undergraduate research scholarship from the Federal University of Pampa.

## Ethics Statement

This study was approved by the Ethics Committee for Research with Humans from the Federal University of Pampa on October 21, 2023 (Reference number: IRB 73202423.8.0000.5323). All participants provided written informed consent prior to their inclusion in the study.

## Consent

Informed consent was obtained from all participants involved in the study.

## Conflicts of Interest

The authors declare no conflicts of interest.

## Supporting information


Supporting Information S1

Supporting Information S2

Supporting Information S3

## Data Availability

The data that support the findings of this study are available from the corresponding author upon reasonable request.
