# Supplementary material for: Acute Foot Warming Enhances Plantar Tactile Sensitivity With Limited Postural Control Effects in Obese Older Adults
Source: Physiother Res Int. 2026 Aug 17;31(4):e70317. doi: 10.1002/pri.70317 (PMC13480721; doi:10.1002/pri.70317)
Supplement: Supplementary file 1 [file PRI-31-e70317-s003.docx]

Supplemental file 2. Mean ± standard deviation foot temperature (°C) measured in the different foot regions in middle-aged adults, non-obese older adults, and obese older adults before (basal) and after (post) the foot warming protocol.

|  | Middle-aged adults | | | Non-obese older adults | | Obese older adults | |
| --- | --- | --- | --- | --- | --- | --- | --- |
|  | | Basal | Post | Basal | Post | Basal | Post |
| Forefoot (ºC) | | 30.0 ± 1.7 | 37.3 ± 1.3* | 28.8 ± 1.8 | 36.2 ± 1.9* | 29.1 ± 1.7 | 36.2 ± 1.9* |
| Midfoot (ºC) | | 29.6 ± 1.7 | 36.8 ± 1.8* | 29.1 ± 1.4 | 36.5 ± 1.4* | 29.0 ± 1.8 | 36.9 ± 1.7* |
| Rearfoot (ºC) | | 27.9 ± 2.3 | 35.4 ± 1.9* | 28.4 ± 1.7 | 35.1 ± 1.5* | 28.7 ± 1.7 | 36.3 ± 2.0* |
| Dorsum (ºC) | | 30.5 ± 1.8 | 36.6 ± 1.6* | 29.7 ± 1.2 | 36.0 ± 1.2* | 30.0 ± 2.0 | 35.7 ± 1.5* |
| ΔT (ºC) | 7.0 ± 0.5 | | | 6.9 ± 0.4 | | 7.0 ± 0.8 | |

* Statistical difference between basal and post foot warming (p < 0.05). For detailed statistical results of foot temperatures, please refer to the supplementary file. ΔT: Indicates the change in skin temperature between pre- and post-intervention time points.
